# Linking Environmental Particulate Matter with Genetic Alterations

**DOI:** 10.1289/ehp.0900830

**Published:** 2009-08

**Authors:** Francesco Cetta, Armand Dhamo, Giuliana Malagnino, Mauro Galeazzi

**Affiliations:** Department of Surgery Research, Doctorate in Oncology and Genetics, University of Siena, Siena, Italy, E-mail: cetta@unisi.it; Department of Clinical Medicine and Immunology, University of Siena, Siena, Italy

In their article, Tarantini et al. (2009) focused on the basic question of mutual relationships between environmental and genetic factors. From a more general point of view, this also involves the question concerning the relative role of “intrinsic toxicity” of xenobiotics and individual susceptibility or host response. In particular, data from epidemiologic and *in vitro* studies must cope with pathologic evidence for pathologists, as well as cause-and effect-relationships for pathophysiologists. In between are inferences and extrapolations on the basis of plausibility.

Tarantini et al. (2009) showed, for the first time in humans, that reactive oxygen species (ROS)—which are considered one of the main cellular stressors generated by PM exposure—may produce genomic hypomethylation and increased expression and activity of inducible nitric oxide synthase (*iNOS*) not only *in vitro*, but in humans exposed to particulate matter (PM). Although this finding is expected, it is a step forward, based on DNA adduct generation produced by polycyclic aromatic hydrocarbons (PAHs) and other PM components, namely transition metals. Alterations of DNA methylation of the promoter is a common finding in environmental-related chronic or cancerous diseases.

Alterations in DNA methylation and iNOS methylation, as observed by Tarantini et al. (2009) in association with exposure to PM < 10 μm in aerodynamic diameter, may represent an initial step in reproducing decreases in global DNA methylation content that are eventually observed in cardiovascular diseases and cancer.

However, pathologists and pathophysiologists are required to interpret more correctly what this means. Subjects with inherited multi tumoral syndromes have a germline mutation, usually a point mutation present in all cells of the body, which determines the occurrence of multiple tumors in the same individual. The occurrence of DNA adducts or mutations in some cells or tissues due to exposure to PAHs or diesel exhaust does not necessarily induce clinically evident outcomes in the future, because each individual is endowed with a wide variety of natural defenses and repair mechanisms that usually overcome every type of DNA damage. Therefore, the first inference to avoid is that DNA adduct formation or alterations in the promoter methylation of a gene causes cancer in the absence of inherited or acquired predisposition (i.e., a point germline mutation of a tumor suppressor gene or an acquired sporadic mutation). In addition, even in patients with inherited multitumoral syndromes (i.e., in subjects with germline mutations of suppressor genes), tumor occurrence (type and severity) is also greatly influenced by epigenetic factors*,* environmental stimuli, or even long-distance catastrophes. We previously demonstrated an increased incidence of papillary thyroid carcinoma in three members of the same familial adenomatous polyposis (FAP) family (i.e., a kindred having a 1061 *APC* gene mutation). This mutation is responsible for FAP, in part as a side effect (long-term–long-distance) after the Chernobyl disaster, thus suggesting a wider than expected impact of environmental disasters in predisposed subjects (Cetta et al. 1997, 2000).

Analogously, chronic exposure to toxic or carcinogenic environmental substances does not elicit the same results in all individuals. The final clinical outcome (cancer, asthma, pulmonary fibrosis, atherosclerosis, or coronary diseases) seems to be less dependent on the toxic potency of the pollutant or of the exposure dose and more on the individual susceptibility of the host (Cetta 2009a, 2009b). This approach should facilitate a better understanding of the < 5% incidence of mesotheliomas in subjects with the same chronic exposure to asbestos, or the absence of health effects in husbands with chronic occupational exposure to asbestos but the occurrence of mesotheliomas in wives with minor indirect exposure from their husbands (Cetta F, unpublished data).

However, acute and chronic inflammation is the first pathological step. The final clinical outcome (e.g. cancer) does not depend on the first DNA adduct formed or a genetic mutation produced by xenobiotics. A long, automaintaining process will start, such as in liver cirrhosis, leading to cancer or chronic alterations as the final result of the interactions between host and the pathogenic agent. This process is greatly influenced by individual susceptibility or resistance. In PM-related diseases, a major role—in addition to the intrinsic toxicity of the xenobiotic—is played by individual host susceptibility and reactivity, similar to what occurs in auto immune or autoinflammatory diseases.
